# Cognitive Mechanisms of Change in Delusions: An Experimental Investigation Targeting Reasoning to Effect Change in Paranoia

**DOI:** 10.1093/schbul/sbu103

**Published:** 2014-07-22

**Authors:** Philippa Garety, Helen Waller, Richard Emsley, Suzanne Jolley, Elizabeth Kuipers, Paul Bebbington, Graham Dunn, David Fowler, Amy Hardy, Daniel Freeman

**Affiliations:** ^1^Department of Psychology, Institute of Psychiatry, King’s College London, London, UK;; ^2^Centre for Biostatistics, Institute of Population Health, The University of Manchester, Manchester Academic Health Science Centre, Manchester, UK;; ^3^Mental Health Sciences Unit, Faculty of Brain Sciences, University College London, London, UK;; ^4^Department of Psychology, University of Sussex, Brighton, UK;; ^5^Department of Psychiatry, Oxford University, Oxford, UK

**Keywords:** jumping to conclusions, belief flexibility, mediators, reasoning biases, psychosis

## Abstract

***Background:*** Given the evidence that reasoning biases contribute to delusional persistence and change, several research groups have made systematic efforts to modify them. The current experiment tested the hypothesis that targeting reasoning biases would result in change in delusions. ***Methods:*** One hundred and one participants with current delusions and schizophrenia spectrum psychosis were randomly allocated to a brief computerized reasoning training intervention or to a control condition involving computer-based activities of similar duration. The primary hypotheses tested were that the reasoning training intervention, would improve (1) data gathering and belief flexibility and (2) delusional thinking, specifically paranoia. We then tested whether the changes in paranoia were mediated by changes in data gathering and flexibility, and whether working memory and negative symptoms moderated any intervention effects. ***Results:*** On an intention-to-treat analysis, there were significant improvements in state paranoia and reasoning in the experimental compared with the control condition. There was evidence that changes in reasoning mediated changes in paranoia, although this effect fell just outside the conventional level of significance after adjustment for baseline confounders. Working memory and negative symptoms significantly moderated the effects of the intervention on reasoning. ***Conclusion:*** The study demonstrated the effectiveness of a brief reasoning intervention in improving both reasoning processes and paranoia. It thereby provides proof-of-concept evidence that reasoning is a promising intermediary target in interventions to ameliorate delusions, and thus supports the value of developing this approach as a longer therapeutic intervention.

## Introduction

Two strands of research form the background to this study: first, the development and evaluation of cognitive behavioral therapy (CBT) for psychosis and secondly, hypotheses about causal mechanisms of delusion formation derived from cognitive models of psychosis. There is good evidence for the effectiveness of CBT for psychosis, summarized in meta-analyses.^1–4^ These are consistent in finding that CBT improves a range of outcomes, including positive symptoms of psychosis, with a small effect size of around 0.2–0.4. However, the modest effects of CBT in meta-analyses indicate a need to improve CBT.^5^ Recent research has also substantially increased our knowledge of cognitive processes likely to contribute to persistence and change in delusions.^6^ However, we have little evidence so far that CBT for psychosis does change the cognitive processes hypothesized to maintain delusions. It has been suggested that in order to achieve improvements in therapy, we must increase our understanding of cognitive mechanisms of symptom change.^7,8^ Manipulations specifically designed to target theoretically derived processes should provide the basis for developing new, more effective methods of intervention, an approach used successfully to improve treatments in emotional disorders (eg, Clark et al^9^) and hallucinations.^10,11^

Cognitive models of delusions propose that their development and persistence is shaped by both emotional and reasoning processes.^6,12^ It is held that reasoning biases distort the appraisal of disturbing anomalous experiences and adverse events: in particular, limited data gathering leads to a rapid acceptance of implausible ideas without consideration of alternative explanations (AE).^12–15^ The current study concerns 2 reasoning biases prominent in people with delusions: (1) a tendency to gather less data than controls to reach a decision, the “jumping to conclusions” bias (JTC); and (2) limitations in belief flexibility, ie, in the “metacognitive process of thinking about one’s own delusional beliefs, changing them in the light of reflection and evidence and generating and considering alternatives.”^6,16^ Poor belief flexibility is common in people with delusions, and is related to, but distinct from, delusional conviction and JTC. Even people with maximal conviction delusions are found to differ in belief flexibility; in one study, one quarter of 110 people expressing 100% delusional conviction concurrently responded that they might be mistaken: thus people can be equally convinced that they are correct in asserting a belief but differ in their relationship to that conviction.^17^ JTC and belief flexibility do not appear to be changed by antipsychotic medication^17,18^ but there is emerging evidence that both phenomena may moderate the response to antipsychotic and psychological treatments: people with a JTC bias or limited belief flexibility show a poorer treatment response.^17,19–22^ Changes in delusions are also associated with changes in data gathering on a JTC task.^23,24^

Given the potential importance of these reasoning biases in delusion change and persistence, several groups have made systematic attempts to modify them.^25–27^ Moritz and colleagues,^28^ have pioneered “metacognitive training” (MCT) for a range of social-cognitive biases, found in psychosis, including JTC. They obtained improvements in delusions in a large randomized controlled trial (RCT), using group training with a broadly defined clinical population, with past or current delusions whose severity was rated as low-moderate.^29^ However, another RCT of group-based MCT with more severe delusions recently found no effect, either on delusions or reasoning processes.^30^ Furthermore in Moritz et al’s study, those in the MCT condition did not experience significantly greater improvements in reasoning than controls receiving cognitive remediation training.

We have developed a brief computerized reasoning training program (the Maudsley Review Training Program), building on Moritz’s development of MCT. However, our program differs in that it targets JTC and belief flexibility more intensively, incorporates material intended to be personally relevant and salient, and is delivered individually rather than in a group format. In 2 pilot studies, data gathering was improved in people with current delusions.^31,32^ In one, delusional conviction also significantly declined: this employed more delusion-relevant (paranoia) training materials, with a stronger focus on enhancing belief flexibility.^32^ Although encouraging, no randomized controlled study has yet convincingly demonstrated that change in reasoning processes as a result of a reasoning intervention accompanies and mediates improvements in delusion outcomes.

Factors affecting the course of psychosis include neurocognitive deficits, particularly working memory and attention. These are related to negative symptoms, poorer functioning, and worse overall outcomes.^33^ An association between JTC and working memory deficits has been demonstrated^34–36^ and, recently, it has been found that JTC is linked to negative symptoms.^36^ It is therefore plausible that impaired working memory and negative symptoms might both negatively moderate the effect of the reasoning intervention on the targeted mediators (reasoning processes) and outcomes.

## Study Aim, Design, and Hypotheses

The current study tested the hypothesis that targeting reasoning biases would result in change in delusions. The study was not a test of therapy delivered at a level of intensity or dose intended to achieve clinically important long-term change; rather, it employed an experimental reasoning intervention in order to achieve short-term change in delusional state, investigating hypothesized relationships between these variables to establish proof-of-concept. In addition to investigating mechanisms of change, we also wished to examine hypothesized moderators of outcomes.

Participants were randomly allocated to a computerized reasoning training intervention or to a control condition involving computer-based activities of similar duration. The primary hypotheses tested were that a reasoning training intervention targeting the JTC reasoning bias and belief flexibility, compared with the control condition, would:

1. Improve data gathering and belief flexibility (hypothesized mediators); and
2. Show improvements in state levels of delusional thinking, specifically state paranoia (outcome);

As secondary analyses, we examined:

3. Whether improvement in delusional outcomes would be mediated by changes in JTC and belief flexibility
4. Whether baseline (preintervention) levels of working memory, negative symptoms, and baseline reasoning biases (JTC and belief flexibility) would negatively moderate response to the intervention.

## Methods

### Participants

We recruited 101 individuals with current delusions and a schizophrenia spectrum disorder from 6 National Health Service (NHS) mental health services in London and Norfolk, UK. The inclusion criteria were: a diagnosis of schizophrenia spectrum disorder (International Classification of Diseases-10, F20-29); a current delusional belief consistently held over 3 months with at least 50% self-rated conviction, and rated as distressing (>0 on a Visual Analog Scale of distress); aged 18–65 years; and fluent in English. Individuals were excluded if they had a primary diagnosis of alcohol or substance dependence, organic syndrome or learning disability, or profound visual impairment. They were also excluded if currently undertaking a course of psychological therapy focused on delusions. The current study was 1 of 2 separate but linked studies, employing randomized experimental designs to test hypothesized mechanisms of change in delusions, using common measures but separate patient samples (Freeman et al, submitted).

### Procedure

The research had been reviewed by an NHS research ethics committee, and all participants provided written informed consent. Assessments were carried out at 4 points. Baseline assessments for the purposes of clinical, diagnostic, and demographic description, and of assessing potential moderators, took approximately 3 h and were completed over at least 2 meetings, depending on the pace participants found comfortable. Following completion of baseline assessments, randomization was carried out, using an independent dedicated randomization service. The premanipulation and first postmanipulation assessments each took an hour, being completed before and after the experimental manipulation. A second postmanipulation assessment was completed after a homework interval, 2 weeks after the first post assessment.

### Experimental and Control Interventions

Those allocated to the experimental manipulation completed the Maudsley Review Training Program together with a researcher (see Waller et al^32^ for a detailed description). This brief interactive computerized intervention aims to provide education on reasoning biases (belief inflexibility and JTC), as well as teaching a number of strategies aimed at reducing these biases. It comprises an educational introduction, then 5 tasks, aimed both at enhancing self-awareness of biases, and also training the key strategies: looking for more evidence and thinking carefully before reaching a definite conclusion; being aware of the impact that mood and past experiences have on our thinking; and generating alternative, less distressing, explanations for events. One of the tasks (picture completion), which encourages data gathering, was adapted from the metacognitive training package of Moritz et al.^28^ The other 4 tasks were all developed especially for the program. Three of the tasks include materials designed to trigger paranoid thinking styles, in order to elicit “hot cognitions” and to teach strategies likely to generalize to participants’ own experiences and paranoid beliefs. All tasks were designed to be interactive and engaging and included simple puzzles, video recordings, and short film clips. Throughout the program, the researcher (a psychology graduate) checked on participants’ understanding, provided further clarification if needed, and prompted discussion of ideas, especially when relating aspects of the program to participants’ own experiences. Overall, the training lasted 1.5–3 h, depending on the amount of discussion and took place over 3 meetings, which included the premanipulation and first postmanipulation assessments. The experimental training also included homework exercises, with a specially designed booklet, supported by 2 researcher-initiated telephone calls, over the 2 weeks between the first and second post manipulation assessments (post 1 and post 2).

The control manipulation was chosen to control for similar activity and attentional task requirements, while being inactive with respect to the targeted reasoning processes. It comprised 3 interactive computer tasks, based on information processing paradigms, designed to take approximately the same time as the experimental training. If participants completed these too quickly, 2 neutral video clips could be shown. The tasks were: McCollough visual after-effects, involving looking at a black and white grid, then rating the visual effect obtained in a range of positions^37^; Kamin blocking – the “Film Stars” task of Jones et al^38^; and a latent inhibition task, based on that of Young et al.^39^ The Control manipulation typically lasted 1.5 h and was completed over 3 meetings, which again included the pre and post 1 assessments. Participants in the control condition also received 2 phone calls in the 2 weeks after the first post manipulation assessment, to check how they were, and how they were finding participation, and to remind them to attend the final (post 2) assessment meeting.

### Measures

#### Symptom Measures (Baseline Assessments).

Psychotic symptoms: The Scale for the Assessment of Positive Symptoms and the Scale for the Assessment of Negative Symptoms (SAPS and SANS).^40,41^ The SAPS and SANS are widely used and well-validated semistructured interviews designed to assess the presence over the past month of positive and negative symptoms associated with schizophrenia. The SAPS covers 35 items, while the SANS covers 25. Both have good psychometric properties. For the current study, the SAPS was used to characterize the delusion subtypes. The ratings were summed to create total scores for the SANS and the SAPS.

Depression and anxiety:

Beck Depression Inventory (BDI-II)^42^ and the Beck Anxiety Inventory (BAI).^43^ The BDI-II is a 21-item self-report instrument for assessing symptoms of depression occurring over the past 2 weeks on a 4-point scale (0–3). The BAI includes 21 items rated on the same 4-point scale, but is assessed over the previous week.

#### Neurocognitive Measures (Completed at Baseline).

Neuropsychological tests: Wechsler Test of Adult Reading (WTAR)^44^ and Wechsler Adult Intelligence Scale (WAIS) III subtest: Digit Span.^45^

Premorbid IQ was estimated using the WTAR, which comprises pronouncing 50 irregularly spelled words. Working memory was assessed by the Digit Span subtest of WAIS III: participants are first read a series of numbers and asked to repeat them in the correct order; the series increases progressively in length until the participant fails. This is then repeated with a different series of numbers, but this time the participant is asked to give the numbers in reverse order. The scores are the series lengths correctly recalled forward, backward, and totaled.

#### State Paranoia Measure (Completed Pre- and Postintervention).

We constructed a latent state paranoia variable from 7 measures tapping state paranoia: We used 6 Visual Analog Scale items assessing ideas of persecution and reference taken from Green et al’s Paranoid Thought Scales^46^: I am being deliberately harmed or upset, there is a conspiracy against me, I am being followed, I am being persecuted, I am feeling under threat from others, and I am being laughed at behind my back. These items were selected as having both high loadings on the persecution or reference subscales and also as representative of a range of key paranoid concerns; they have good internal reliability (Cronbach’s alpha = .86).^47^ For each item, participants rated how they were feeling “right now” from 0 (not at all) to 100 (totally). Finally a seventh item assessed delusional conviction on a self-rated scale of 0–100. Factor loadings for state paranoia were estimated for a standardized latent factor at the premanipulation assessment, and then used to calculate the factor scores at the 2 postmanipulation assessments.

#### Reasoning Measures (Completed Pre- and Postintervention).

Reasoning: JTC–probabilistic reasoning task.^16^ Two computerized versions of the probabilistic reasoning (Beads) task, with 85:15 (easy) and 60:40 (difficult) task ratios, were used. For example, for the easy version, one jar had 85 orange beads and 15 black beads, while the other had 15 orange beads and 85 black beads. Participants were shown the 2 jars, and told that one of the jars would be selected at random by the computer and that beads would be drawn from and replaced in the selected jar. After each bead was drawn, participants were asked if they would like to see more beads (ie, if they would like more information) or if they could say, with certainty, from which of the jars the beads were being drawn. Once a bead had been drawn, it was shown at the bottom of the screen, thereby providing a memory aid. The key variable was the number of beads requested by the participant before making a decision. We report both the mean number of beads drawn and a dichotomous variable (Yes/No), where Yes was classified as requesting 2 or fewer beads.

Belief flexibility: Maudsley Assessment of Delusions Scale (MADS)^48^ and the Explanations of Experiences (EoE) measure.^49^ Two MADS items were used to measure aspects of belief flexibility (the possibility of being mistaken [PM], and the reaction to hypothetical contradiction). The evidence for the delusion cited by participants is sensitively discussed, and they are asked whether it is at all possible for them to be mistaken about their delusional belief. The interviewer then asks how they would react in a hypothetical situation if some new evidence were to contradict the grounds for the delusion. If they report that this would alter their level of belief in any way, this is recorded as belief flexibility. The EoE measure is a structured interview designed to assess whether people can envisage AE for the evidence cited for their delusion. Once the evidence for the delusion is established, they are asked “Can you think of any other explanations for the experiences that you have described? Are there any other reasons — other than [the delusional belief] — that could possibly account for these experiences even if you think they are very unlikely?” The generation of any AE is also taken as a measure of belief flexibility. All 3 belief flexibility measures are rated as present or absent, but the possibility of being mistaken is also scored on a scale of 0–100 (from “not at all” to “totally”).^32^ In a factor analysis of a sample of 300 people with psychosis, these 3 measures formed a coherent and stable belief flexibility factor.^17^

#### Other State Symptom Measures (Completed Pre- and Postintervention).

Hallucinations Visual Analog Scales: Participants reporting any experience of hallucinations were asked to state the main, or most distressing experience and rated its frequency and distress “right now,” from 0 (not at all) to 100 (totally). Anxiety and Depression Visual Analog Scales: Participants were asked to rate how “anxious” and how “depressed” they were feeling “right now,” from 0 (not at all) to 100 (totally).

### Analysis

All analyses were carried out using Stata version 13.1.^50^ In a conventional intention-to-treat (ITT) approach, ANCOVA was used to evaluate the effect of the randomization condition on the outcome (paranoia) and, separately, the putative mediators (JTC and belief flexibility) as dependent variables. We allowed for center and the baseline measures as covariates in these models. Mediation analysis was performed using the methods of Baron and Kenny^51^ and as extended in Valeri and Vanderweele,^52^ to investigate direct and indirect effects of the experimental manipulation on paranoia. In addition to the previous ITT models, this involved regressing paranoia on the randomized condition and the separate mediators in a linear model. The effect of randomized condition on the mediator and the effect of the mediator on paranoia are multiplied to estimate the indirect effect. Since a variable can only be a mediator if there is a significant effect of randomized condition on the mediator, mediation analysis was only performed when there was a significant ITT effect on the mediators. We performed the mediation analysis with and without adjustment for baseline covariates in all 3 models. Estimates of the direct and indirect effects can be biased, even in randomized trials, when there are unmeasured confounders between the mediator and outcome.^53^ By including baseline measures of the outcome and mediators in the regression models, we attempt to control for these as potential confounders in order to add robustness to our analysis. Finally, we tested whether any of the ITT effects might be subject to moderation by baseline (prerandomization) covariates by including interactions between the baseline covariates and randomization in the models. The results presented here are of complete cases, so that patients with missing outcomes or mediator values are not included in the analysis; we indicate the numbers included in our results. This approach assumes that, conditional on the baseline covariates and randomization, the missing outcomes and mediators are missing at random.

## Results

### Participants: Demographic, Clinical, and Neurocognitive Data

The study was powered for the primary outcome on a sample of 100 participants; we recruited to this target, and the final sample comprised 101 participants. Of these, 61 were male and 40 female. Their mean age was 41.6 years (SD = 11.0) and they had been diagnosed for 14.7 years (SD = 9.9). Sixty two (61%) were recorded as of white ethnicity, while 24 (24%) were black (African or Caribbean), and the remaining 15 (15%) Asian or other. Diagnoses were derived using OPCRIT from SCAN interviews (Schedules for Clinical Assessment in Neuropsychiatry)^54^ and were as follows: schizophrenia 89 (88%); delusional disorder 3 (3%); schizoaffective disorder 6 (6%); and other nonorganic psychotic disorders 3 (3%). The participants were nearly all currently taking antipsychotic medication (94 of 101). All completed the SAPS and all had delusions of at least moderate (score 3) severity, with high levels of delusional conviction: 50% reported 100% conviction and 80% of the total sample reported conviction at 75% and above. Eighty-eight percent had at least one delusion that was persecutory in content, while the remainder all had delusions with content related to persecutory concerns, mainly in the context of ideas of reference. The other delusion types recorded, most frequently co-occurring with persecutory delusions, were: 69 Reference, 51 Mind being read, 26 Thought Insertion, 23 Thought Broadcast, 20 Being controlled, 20 Religious, 17 Somatic, 13 Grandiose, 9 Thought withdrawal, and 8 Guilt. Participants had moderate levels of depression (BDI mean 22.1; SD = 12.4) and of anxiety (BAI mean 24.9; SD = 13.0).

Table 1 shows summary statistics, for each randomized group, for the baseline neurocognitive and negative symptom measures, that were hypothesized to be moderators of effects. The SAPS (positive symptoms) scores are also shown.

**Table 1.** Summary Baseline Values of Neurocognitive and Symptom Scores

Measure	Experimental Group				Control Group			
	Mean	SD	Range	*N*	Mean	SD	Range	*N*
Wechsler Test of Adult Reading	87.09	18.37	53–124	47	90.45	19.16	50–120	49
Digit span (raw total)	15.47	4.74	8–27	51	15.48	3.90	7–23	50
Digit span (forward)	9.78	2.86	5–16	51	9.88	2.55	6–14	50
Digit span (backward)	5.69	2.24	2–11	51	5.60	2.01	1–10	50
Negative symptoms (SANS total)	9.68	3.68	3–18	50	10.31	3.99	2–18	49
Positive symptoms (SAPS total)	8.82	2.73	4–15	51	9.1	2.57	4–15	50

*Note:* SANS, Scale for the Assessment of Negative Symptoms; SAPS, Scale for the Assessment of Positive Symptoms.

### Outcome and Reasoning (Mediators) Summary Data

Table 2 shows summary statistics at each time point for the state paranoia outcome measure, and the reasoning variables, for each randomized group separately. Four participants dropped out of the study between baseline and the start of the intervention and a further 4 dropped out over the course of the 2-week intervention period (from pre- to post 2), 2 from each condition.

**Table 2.** Summary Outcome (Paranoia) and Hypothesized Reasoning (Mediators) Data

Measure	Time	Experimental Group				Control Group			
		Mean	SD	Range	*N*	Mean	SD	Range	*N*
Outcome									
State paranoia factor	Pre	−0.13	0.84	−1.1, 2.3	50	0.10	0.99	−1.2, 2.3	46
	Post 1	−0.11	0.98	−1.1, 2.1	47	0.10	1.02	−1.1, 2.1	43
	Post 2	−0.23	0.77	−1.1, 1.9	47	0.22	1.03	−1.1, 2.2	45
Mediator									
Possibility of being mistaken, %	Pre	18.0	26.2	0–100	50	18.9	26.5	0–100	46
	Post 1	23.4	29.1	0–100	48	19.4	28.0	0–100	47
	Post 2	24.8	28.5	0–90	48	15.5	22.7	0–90	44
Number of beads—85/15	Pre	4.5	4.5	1–20	50	4.1	3.6	1–19	47
	Post 1	6.3	5.1	1–20	48	4.0	3.7	1–20	47
	Post 2	7.0	5.9	1–20	48	5.5	5.3	1–20	45
Number of beads—60/40	Pre	7.1	6.0	1–20	50	7.0	5.1	1–20	47
	Post 1	8.6	5.8	1–20	48	6.9	5.2	1–20	47
	Post 2	9.0	6.4	1–20	48	6.8	4.6	1–18	45
			**N**		**%**		**N**		**%**
JTC 85:15	Pre	No	34		68.0		28		59.6
		Yes	16		32.0		19		40.4
	Post 1	No	39		81.3		25		53.2
		Yes	9		19.7		22		46.8
	Post 2	No	37		77.1		31		68.9
		Yes	11		22.9		14		31.1
JTC 60:40	Pre	No	34		68.0		35		74.5
		Yes	16		32.0		12		25.5
	Post 1	No	41		85.4		36		76.6
		Yes	7		14.6		11		23.4
	Post 2	No	37		77.1		36		80.0
		Yes	11		22.9		9		20.0
Possibility of being mistaken, Y/N	Pre	No	26		52.0		29		61.7
		Yes	24		48.0		18		38.3
	Post 1	No	23		47.9		25		53.2
		Yes	25		52.1		22		46.8
	Post 2	No	15		31.3		25		56.8
		Yes	33		68.7		19		43.2
Alternative explanations	Pre	No	39		78.0		37		78.7
		Yes	11		22.0		10		21.3
	Post 1	No	33		68.8		36		76.6
		Yes	15		31.2		11		23.4
	Post 2	No	27		56.3		33		73.3
		Yes	21		43.7		12		26.7
Hypothetical contradiction	Pre	No	34		69.4		35		74.5
		Yes	15		30.6		12		25.5
	Post	No	31		64.6		33		70.2
		Yes	17		35.4		14		29.8
	Post 2	No	30		62.5		28		63.6
		Yes	18		37.5		16		36.4

*Note:* JTC, jumping to conclusions.

### Primary ITT Analysis

Table 3 shows the ITT effects (the differences between the groups) on state paranoia, and on the hypothesized mediators, at each time point separately and after adjustment for baseline values of the measure and center. The primary end point for the paranoia outcome is at post 2, after the homework exercises had been completed. There was a significant effect on paranoia, with the intervention group showing a reduction (improvement) compared with the control group at post 2, with an effect size of −0.36. Both time points—immediately after the training and after the homework period—are of interest for the investigation of effects on mediators. There were significant effects (improved data gathering and more flexibility in reasoning, greater in the intervention than the control) mainly at post 2 on both the beads tasks and 2 on the belief flexibility measures (PM and AE). The effect size on the number of beads 85/15 was 0.31 at post 1, while that for 60/40 was 0.31 at post 1, and 0.40 at post 2. The effect size for probability of being mistaken at post 2 was 0.35. There were no significant effects of the intervention at either time point on hallucinations, anxiety, or depression.

**Table 3.** Effect of Experimental Group Compared With Control Group on Outcome and Mediator Measures

Measure	Time	Effect	SE	*P* Value	95% CI	*N*
Outcome						
State paranoia factor	Post 1	−0.00	0.13	.985	−0.26, 0.26	90
	Post 2	−0.36	0.16	.028	−0.67, −0.04	92
Mediators						
Beads—85/15	Post 1	1.97	0.73	.008	0.52, 3.43	95
	Post 2	1.17	1.03	.262	−0.89, 3.22	93
Beads—60/40	Post 1	1.68	0.82	.045	0.04, 3.32	95
	Post 2	1.86	0.87	.035	0.13, 3.59	93
Possibility of being mistaken, yes/no	Post 1	OR = 1.09	0.52	.850	0.43, 2.79	95
	Post 2	OR = 4.10	2.28	.011	1.38, 12.17	92
Alternative explanations	Post 1	OR = 2.24	1.48	.222	0.62, 8.17	95
	Post 2	OR = 2.90	1.58	.051	1.00, 8.45	93
Possibility of being mistaken, %	Post 1	4.02	5.16	.438	−6.23, 14.28	94
	Post 2	9.71	5.01	.056	−0.25, 19.68	94
Hypothetical contradiction	Post 1	OR = 1.28	0.65	.621	0.48, 3.44	95
	Post 2	OR = 0.96	0.51	.937	0.34, 2.71	92
Other symptom states						
Hallucination frequency–VAS	Post 1	−4.80	19.34	.805	−43.53, 33.94	63
	Post 2	−10.85	20.54	.600	−52.02, 30.32	61
Hallucination Distress–VAS	Post 1	−8.01	6.30	.209	−20.62, 4.60	64
	Post 2	2.95	5.90	.619	−14.77, 8.87	62
Anxiety–VAS	Post 1	−0.47	5.05	.927	−10.51, 9.58	93
	Post 2	−1.00	6.29	.874	−13.50, 11.50	93
Depression–VAS	Post 1	1.48	5.41	.785	−9.27, 12.24	93
	Post 2	3.13	5.92	.599	−8.64, 14.89	93

*Note:* VAS, Visual Analog Scale.

### Mediation Analysis

We employed the state paranoia factor at post 2 as the outcome for the mediation analysis, using only those measures significant at either time point as mediators (table 3: for this purpose, *P* < .1). The analyses present results with and without adjustment for the pretest values for paranoia and all the putative mediators, together with recruitment center, as covariates (table 4). In the adjusted analysis, there was evidence of partial mediation for the reduction in paranoia by belief flexibility (approximately 40% for PM, as a dichotomous variable) and, to a lesser extent (21%), for AE. The evidence of mediation by belief flexibility (PM) fell just outside the conventional level of significance (*P* = .057). In the unadjusted analysis, we found a significant indirect effect through belief flexibility PM (*P* = .042). There was no evidence that changes in JTC mediated a reduction in paranoia.

**Table 4.** Statistical Mediation Analysis for State Paranoia Within Each Mediator, the Top Row Shows the Adjusted Analysis, the Bottom Row Shows the Unadjusted Analysis

Mediator	Total Effect, Effect (SE), *P* Value	Direct Effect, Effect (SE), *P* Value	Mediated Effect, Effect (SE), *P* Value	Proportion Mediated, %	*N*
Beads 85/15 at post 1	−0.35 (0.16), .027	−0.33 (0.16), .040	−0.02 (0.04), .591	5.7	94
	−0.46 (0.19), .015	−0.39, (0.19), .042	−0.07 (0.06), .232	15.2	94
Beads 60/40 at post 1	−0.35 (0.16), .026	−0.36 (0.16), .027	0.00 (0.03), .889	0.0	94
	−0.45 (0.19), .018	−0.42, (0.19), .030	−0.04 (0.04), .351	8.9	94
Beads 60/40 at post 2	−0.36 (0.16), .025	−0.33 (0.16), .041	−0.02 (0.04), .512	5.5	93
	−0.46 (0.19), .016	−0.42, (0.19), .031	−0.04 (0.04), .331	8.7	93
PM % at post 2	−0.39 (0.16), .012	−0.34 (0.16), .031	−0.06 (0.04), .197	15.4	91
	−0.46 (0.19), .016	−0.35, (0.18), .056	−0.11 (0.07), .126	23.9	91
PM—yes/no at post 2	−0.44 (0.20), .025	−0.27 (0.15), .077	−0.17 (0.08), .057	38.6	92
	−0.47 (0.22), .029	−0.31 (0.19), .097	−0.16 (0.08), .042	34.0	92
AE—yes/no at post 2	−0.38 (0.21), .066	−0.30 (0.16), .060	−0.08 (0.06), .182	21.1	93
	−0.45 (0.23), .056	−0.35 (0.18), .058	−0.10 (0.07), .130	22.2	93

*Note:* AE, alternative explanations; PM, possibility of being mistaken.

### Moderation Analysis

We considered whether the effect our intervention on the significant mediators and on the paranoia outcome was moderated by the following variables assessed at baseline: reasoning biases, premorbid IQ, working memory, and negative symptoms. There was no significant moderation of the mediators by any of the pretest (baseline) reasoning variables or by premorbid IQ. Thus, the intervention is effective in improving reasoning, regardless of pretest biases in data gathering (JTC), belief flexibility, and levels of premorbid IQ.

The results of moderation by working memory and negative symptoms on the effects of the intervention on reasoning are given in table 5, based on *P* levels of <.1. Digit span (working memory) significantly moderated the effect of the intervention on JTC and on belief flexibility (PM); when analyzed separately, this was significant for digit span forward but not backward. Better working memory enhanced the effects of the intervention on reasoning. Negative symptoms significantly moderated the effect of the intervention on PM (both expressed as percentage [*P* = .046], and, at trend level, when measured dichotomously, *P* = .070). The effect of the intervention in improving belief flexibility was reduced as negative symptom scores increase. There was, however, no significant moderation of the paranoia outcome by any of the hypothesized moderator variables.

**Table 5.** Statistical Moderation Analysis for Mediator (Reasoning) Outcomes at Post 2 Timepoint

Moderator	Mediator at Post 2	Interaction Effect	SE	*P* Value	*N*
Digit span	Number of beads 60/40	0.46	0.20	.022	93
Digit span	PM (yes/no)	1.27	0.17	.073	92
Digit span forward	PM (yes/no)	1.58	0.34	.036	92
Digit span forward	Number of beads 60/40	0.86	0.31	.008	93
SANS	PM%	−2.64	1.30	.046	91
SANS	PM (yes/no)	0.75	0.12	.070	92

*Note:* PM, possibility of being mistaken; SANS, Scale for the Assessment of Negative Symptoms.

## Discussion

This study has a novel combination of features. It used an experimental design in a clinical population of people with distressing, strongly held delusions, it randomized between 2 conditions matched for duration and contact, and it has demonstrated the effectiveness of a brief reasoning intervention in improving both reasoning processes and paranoia. It thereby provides proof-of-concept evidence that reasoning is a promising intermediary target in interventions to ameliorate delusions, and thus supports the potential value of this approach.^27,28,55^

As assessed by most measures, the intervention, despite its brevity, significantly affected reasoning, our putative mediator. The effects on data gathering were seen immediately after training, while belief flexibility only changed significantly after the 2-week homework exercises and generalization. The intervention also significantly affected the targeted outcome of paranoia. This effect occurred, as expected, after the period of homework exercises. We knew that training on its own would be insufficient. We actively encouraged (though did not formally monitor) homework completion in 2 phone calls. Accordingly throughout the intervention, we emphasized to participants the need to generalize the learning from the training to real life situations. The focus in both training and homework exercises was on exploring how people come to decisions and make sense of their everyday experiences. We tried to encourage awareness of reasoning processes in participants and to help them identify and, where appropriate, inhibit rapid, automatic reasoning (“type one” reasoning) and engage in more analytical or controlled reasoning (“type two”).^56–58^

Training also aimed to help participants consider the role of their emotions in thinking processes and to engage “hot cognitions” in order to develop the ability to enlist more reflective analytic reasoning processes under these everyday conditions. This is as recommended by van Oosterhout et al,^30^ who also comment on the potential limitations of a group educational approach of metacognitive training and the importance of arousing personal emotional meanings and using homework exercises to support generalization and change.

Our results indicated that the reduction in paranoia was partially mediated by improvements in reasoning. The study was powered on ITT changes in the primary outcome and in the reasoning biases and was consequently somewhat underpowered for the mediation analysis, but the pattern of results is consistent with one aspect of belief flexibility (awareness of the PM) explaining a reasonably substantial proportion of change in paranoia. In the more stringent, adjusted analyses, this finding fell just outside the conventional level of statistical significance. However, we conclude that this study provides reasonably convincing evidence of a mechanistic effect because: we deliberately used an intervention targeted at a putative mediator (reasoning processes), rather than the outcome (paranoid thoughts); and the mediator was identified by prior theory and with preliminary evidence that attributes of reasoning predicted change in delusion.^6,20,21^ Thus, a causal effect of state paranoia on belief flexibility is unlikely.

This study therefore supports the proposition that targeting belief flexibility may be an effective strategy for intervention with delusions. As well as a lack of flexibility in relation to their own delusions, people with delusions also display more general deficits in belief flexibility and analytical reasoning.^58–60^ Training people to become more aware of and flexible in their general thinking style and to consider that they might be mistaken appears to lead to changes in appraisals of everyday experiences which trigger state paranoia. Moritz and colleagues,^28^ in their recent review, also emphasize the role of “sowing the seeds of doubt” and propose that MCT works through encouraging “*patients to be less confident in their judgments* [our emphasis] and to seek more evidence when little information is available.”

The hypothesis that JTC is a mediator was, however, not supported, counter to expectation. Given that we did find an effect on JTC, but not as a mediator, it might be that learning the general skill of increasing data gathering to support everyday decision making is not relevant to paranoia. In this study, a relatively low proportion of participants showed the JTC bias at baseline in the experimental group (32%); therefore, we had unexpectedly limited power to investigate this hypothesis. We examined the data further to explore this. In the intervention group, those without the bias at both time points and also those who improved in data gathering, both showed reductions in paranoia, whereas those who continued to JTC at both time points did not change in paranoia. It therefore remains possible that JTC mediates changes in paranoia, but only in a subgroup. More studies are needed to clarify the mechanistic role of JTC in delusion changes and prevalence of the JTC bias by delusion subtype.^61^

The effects of the intervention on reasoning were not moderated either by premorbid IQ by baseline reasoning biases. Whereas we previously found that those with more marked reasoning biases responded less well to the training,^31^ we have modified and extended the intervention. This appears to have succeeded in extending its effectiveness to those with strong biases and those with a lower IQ. However, other variables do moderate the effects of the intervention on reasoning changes: people with better working memory and less in the way of negative symptoms responded better to the intervention. Future research should investigate further why the effects were apparent on digit span forward (the maximum length of numbers achieved), which reflects working memory capacity, rather than on digit span backward (the maximum length of numbers given in reverse), which additionally reflects the ability to manipulate items in working memory. “Type two” reasoning, which the intervention specifically aims to enhance, requires working memory capacity^57^ and thus our finding that this moderates outcomes is consistent. Negative symptoms involve both motivational and cognitive capacities. It would be useful to investigate how these different aspects of negative symptoms might contribute to the effects. We conclude that this therapy might be developed further to compensate for both working memory deficits, and negative symptoms.

The study had a number of limitations. The measures of belief flexibility employed related specifically to the main paranoid belief; future research could be improved by incorporating more general self-report and performance assessments of belief flexibility. Although there are some existing self-report scales assessing cognitive flexibility and psychosis-related cognitive biases (eg, Dennis and Vander Wal^62^, Peters et al^63^), the relationship of such measures to delusional belief flexibility has not yet been established, and further work is warranted to establish this. This may illuminate which components of belief flexibility are critical to paranoid thinking and provide the basis for investigating further the causal role of belief flexibility in paranoia. Secondly, we were also somewhat underpowered in relation to the mediation analysis, as discussed. The mediation analysis also assumes that there are no unmeasured confounders between the mediator and outcome, which is possible since both are measured postrandomization. We attempted to control for some confounders by including baseline measures as covariates in the mediation analysis; however, we cannot rule out the presence of further unmeasured confounders influencing the results. Thirdly, assessments of outcome were conducted by independent assessors, but they were not blind to treatment condition. Finally, our primary outcome measure assessed current paranoid state, and although it incorporated a measure of conviction, the latter did not change significantly. The study was not test of a therapy: it was designed as a proof-of-concept experiment, delivered by graduate researchers, not therapists, and the intervention was brief: nevertheless it had an effect size of -−0.36 on paranoia. Longer term, clinically important, and more wide-ranging benefits, including larger effects on delusional conviction, preoccupation, and distress, require a more intensive therapeutic intervention, with trained therapists. Taking account of the findings of the current study, we are currently extending the intervention as a course of 8 sessions, to incorporate a greater number and range of experiential, self-monitoring, and homework exercises, and piloting it, prior to testing in a RCT.

## Funding

Wellcome Trust project grant (085396). P.G. and E.K. are supported by the National Institute for Health Research Biomedical Research Centre for Mental Health at the South London, Maudsley NHS Foundation Trust, and King’s College London. D.F. is supported by a UK Medical Research Council (MRC) Senior Clinical Fellowship. R.E. and G.D. received research funding from the UK MRC (G0900678) and R.E. a MRC Career Development Award (G0802418).

## References

[1] Wykes T Steel C Everitt B Tarrier N Cognitive behavior therapy for schizophrenia: effect sizes, clinical models, and methodological rigor. Schizophr Bull. 2008;34:523–537. 17962231 10.1093/schbul/sbm114 PMC2632426

[2] National Institute of Clinical Excellence (NICE). Core interventions in the treatment and management of schizophrenia in primary and secondary care. London: NICE; March 2009.

[3] National Institute of Clinical Excellence (NICE). Psychosis and Schizophrenia in adults. The NICE guideline on Treatment and Management (full guideline). February 2014 http://guidance.nice.org.uk/CG178.

[4] Turner DT van der Gaag M Karyotaki E Cuijpers P Psychological interventions for psychosis: a meta-analysis of comparative outcome studies. Am J Psychiatry. 2014;171:523–538.. 24525715 10.1176/appi.ajp.2013.13081159

[5] Kingdon D A golden age of discovery. Br J Psychiatry. 2013;202:394–395. 23732932 10.1192/bjp.bp.112.125039

[6] Garety PA Freeman D The past and future of delusions research: from the inexplicable to the treatable. Br J Psychiatry. 2013:327–333. 24187067 10.1192/bjp.bp.113.126953

[7] Kraemer HC Wilson GT Fairburn CG Agras WS Mediators and moderators of treatment effects in randomized clinical trials. Arch Gen Psychiatry. 2002;59:877–883. 12365874 10.1001/archpsyc.59.10.877

[8] Freeman D Improving cognitive treatments for delusions. Schizophr Res. 2011;132:135–139. 21907546 10.1016/j.schres.2011.08.012

[9] Clark DM Ehlers A Hackmann A Cognitive therapy versus exposure and applied relaxation in social phobia: a randomized controlled trial. J Consult Clin Psychol. 2006;74:568–578. 16822113 10.1037/0022-006X.74.3.568

[10] Trower P Birchwood M Meaden A Byrne S Nelson A Ross K Cognitive therapy for command hallucinations: randomised controlled trial. Br J Psychiatry. 2004;184:312–320. 15056575 10.1192/bjp.184.4.312

[11] Birchwood M Peters E Tarrier N A multi-centre, randomised controlled trial of cognitive therapy to prevent harmful compliance with command hallucinations. BMC Psychiatry. 2011;11:155. 21961763 10.1186/1471-244X-11-155 PMC3191332

[12] Freeman D Garety PA Kuipers E Fowler D Bebbington PE A cognitive model of persecutory delusions. Br J Clin Psychol. 2002;41:331–347. 12437789 10.1348/014466502760387461

[13] Garety PA Kuipers E Fowler D Freeman D Bebbington PE A cognitive model of the positive symptoms of psychosis. Psychol Med. 2001;31:189–195. 11232907 10.1017/s0033291701003312

[14] Bentall RP Corcoran R Howard R Blackwood N Kinderman P Persecutory delusions: a review and theoretical integration. Clin Psychol Rev. 2001;21:1143–1192. 11702511 10.1016/s0272-7358(01)00106-4

[15] van der Gaag M A neuropsychiatric model of biological and psychological processes in the remission of delusions and auditory hallucinations. Schizophr Bull. 2006;32(suppl 1):S113–S122. 16905635 10.1093/schbul/sbl027 PMC2632542

[16] Garety PA Freeman D Jolley S Reasoning, emotions, and delusional conviction in psychosis. J Abnorm Psychol. 2005;114:373–384. 16117574 10.1037/0021-843X.114.3.373

[17] So SH Freeman D Dunn G Jumping to conclusions, a lack of belief flexibility and delusional conviction in psychosis: a longitudinal investigation of the structure, frequency, and relatedness of reasoning biases. J Abnorm Psychol. 2012;121:129–139. 21910515 10.1037/a0025297 PMC3283358

[18] Peters E Garety P Cognitive functioning in delusions: a longitudinal analysis. Behav Res Ther. 2006;44:481–514. 15913544 10.1016/j.brat.2005.03.008

[19] Garety P Fowler D Kuipers E London-East Anglia randomised controlled trial of cognitive-behavioural therapy for psychosis. II: Predictors of outcome. Br J Psychiatry. 1997;171:420–426. 9463599 10.1192/bjp.171.5.420

[20] Menon M Mizrahi R Kapur S ‘Jumping to conclusions’ and delusions in psychosis: relationship and response to treatment. Schizophr Res. 2008;98:225–231. 17897811 10.1016/j.schres.2007.08.021

[21] So SH Peters ER Swendsen J Garety PA Kapur S Changes in delusions in the early phase of antipsychotic treatment - an experience sampling study. Psychiatry Res. 2014;215:568–573. 24412352 10.1016/j.psychres.2013.12.033

[22] Dudley R Daley K Nicholson M ‘Jumping to conclusions’ in first-episode psychosis: a longitudinal study. Br J Clin Psychol. 2013;52:380–393. 24117911 10.1111/bjc.12023

[23] Woodward TS Mizrahi R Menon M Christensen BK Correspondences between theory of mind, jumping to conclusions, neuropsychological measures and the symptoms of schizophrenia. Psychiatry Res. 2009;170:119–123. 19906438 10.1016/j.psychres.2008.10.018

[24] Sanford N Woodward TS Lecomte T Leclerc C Wykes T Change in jumping to conclusions linked to change in delusions in early psychosis. Schizophr Res. 2013;147:207–208. 23561297 10.1016/j.schres.2013.02.042

[25] Moritz S Vitzthum F Randjbar S Veckenstedt R Woodward TS Detecting and defusing cognitive traps: metacognitive intervention in schizophrenia. Curr Opin Psychiatry. 2010;23:561–569. 20683332 10.1097/YCO.0b013e32833d16a8

[26] Balzan RP Delfabbro PH Galletly CA Woodward TS Metacognitive training for patients with schizophrenia: preliminary evidence for a targeted, single-module programme. Aust N Z J Psychiatry. Published online October 24, 2013. 10.1177/0004867413508451 10.1177/0004867413508451 24159051

[27] Warman DM Martin JM Lysaker P Jumping to conclusions and delusions: the impact of discussion of the bias on the bias. Schizophr Res. 2013;150:575–579. 24091035 10.1016/j.schres.2013.09.003

[28] Moritz S Andreou C Schneider BC Sowing the seeds of doubt: a narrative review on metacognitive training in schizophrenia. Clin Psychol Rev. 2014;34:358–366. 24866025 10.1016/j.cpr.2014.04.004

[29] Moritz S Veckenstedt R Bohn F Complementary group Metacognitive Training (MCT) reduces delusional ideation in schizophrenia. Schizophr Res. 2013;151:61–69. 24183707 10.1016/j.schres.2013.10.007

[30] van Oosterhout B Krabbendam L de Boer J Metacognitive group training for schizophrenia spectrum patients with delusions: a randomized controlled trial. Psych Med. 2014. 10.1017/S0033291714000555 10.1017/S0033291714000555 25066223

[31] Ross K Freeman D Dunn G Garety P A randomized experimental investigation of reasoning training for people with delusions. Schizophr Bull. 2011;37:324–333. 19520745 10.1093/schbul/sbn165 PMC3044626

[32] Waller H Freeman D Jolley S Dunn G Garety P Targeting reasoning biases in delusions: a pilot study of the Maudsley Review Training Programme for individuals with persistent, high conviction delusions. J Behav Ther Exp Psychiatry. 2011;42:414–421. 21481815 10.1016/j.jbtep.2011.03.001 PMC3145959

[33] Joyce E Huddy V Defining the cognitive impairment in schizophrenia. Psychol Med. 2004;34:1151–1155. 15697041 10.1017/s0033291704003472

[34] Broome MR Johns LC Valli I Delusion formation and reasoning biases in those at clinical high risk for psychosis. Br J Psych. 2007;191:s38–s42. 10.1192/bjp.191.51.s38 18055936

[35] Garety P Joyce E Jolley S Neuropsychological functioning and jumping to conclusions in delusions. Schizophr Res. 2013;150:570–574. 24075604 10.1016/j.schres.2013.08.035 PMC3824078

[36] Freeman D Startup H Dunn G Understanding jumping to conclusions in patients with persecutory delusions: working memory and intolerance of uncertainty. Psych Med. 2014. 10.1017/S0033291714000592 25066636

[37] Hodgekins JM Unpub. PhD thesis. The nature of schizotypal symptoms and social recovery in psychosis. University of East Anglia; 2009.

[38] Jones SH Hemsley D Ball S Serra A Disruption of the Kamin blocking effect in schizophrenia and in normal subjects following amphetamine. Behav Brain Res. 1997;88:103–114. 9401714 10.1016/s0166-4328(97)02312-7

[39] Young AM Moran PM Joseph MH The role of dopamine in conditioning and latent inhibition: what, when, where and how? Neurosci Biobehav Rev. 2005;29:963–976. 16045987 10.1016/j.neubiorev.2005.02.004

[40] Andreasen NC Scale for the Assessment of Positive Symptoms (SAPS). Iowa City, IA: University of Iowa; 1984a.

[41] Andreasen NC Scale for the Assessment of Negative Symptoms (SANS). Iowa City, IA: University of Iowa; 1984b.

[42] Beck AT Steer RA Brown GK BDI-II Manual. San Antonio, TX: The Psychological Corporation; 1996.

[43] Beck AT Epstein N Brown G Steer RA An inventory for measuring clinical anxiety: psychometric properties. J Consult Clin Psychol. 1988;56:893–897. 3204199 10.1037//0022-006x.56.6.893

[44] Wechsler D Wechsler Test of Adult Reading. London: Psychological Corporation; 2001.

[45] Wechsler D Wechsler Adult Intelligence Scale – Third Edition WAIS III. San Antonio, TX: Psychological Corporation; 1997

[46] Green CE Freeman D Kuipers E Measuring ideas of persecution and social reference: the Green *et al*. Paranoid Thought Scales (GPTS). Psychol Med. 2008;38:101–111. 17903336 10.1017/S0033291707001638

[47] Freeman D Dunn G Fowler D Current paranoid thinking in patients with delusions: the presence of cognitive-affective biases. Schizophr Bull. 2013;39:1281–1287. 23223342 10.1093/schbul/sbs145 PMC3796079

[48] Wessely S Buchanan A Reed A Acting on delusions. I: Prevalence. Br J Psychiatry. 1993;163:69–76. 10.1192/bjp.163.1.69 8353703 10.1192/bjp.163.1.69

[49] Freeman D Garety PA Fowler D Kuipers E Bebbington PE Dunn G Why do people with delusions fail to choose more realistic explanations for their experiences? An empirical investigation. J Consult Clin Psychol. 2004;72:671–680. 15301652 10.1037/0022-006X.72.4.671

[50] StataCorp. Stata Statistical Software: Release 13. College Station, TX: StataCorp LP; 2013.

[51] Baron RM Kenny DA The moderator-mediator variable distinction in social psychological research: conceptual, strategic, and statistical considerations. J Pers Soc Psychol. 1986;51:1173–1182. 3806354 10.1037//0022-3514.51.6.1173

[52] Valeri L Vanderweele TJ Mediation analysis allowing for exposure-mediator interactions and causal interpretation: theoretical assumptions and implementation with SAS and SPSS macros. Psychol Methods. 2013;18:137–150. 23379553 10.1037/a0031034 PMC3659198

[53] Emsley R Dunn G White IR Mediation and moderation of treatment effects in randomised controlled trials of complex interventions. Stat Methods Med Res. 2010;19:237–270. 19608601 10.1177/0962280209105014

[54] World Health Organisation. Schedules for Clinical Assessment in Neuropsychiatry (SCAN). Geneva: WHO; 1992.

[55] Lincoln TM Ziegler M Mehl S Rief W The jumping to conclusions bias in delusions: specificity and changeability. J Abnorm Psychol. 2010;119:40–49. 20141241 10.1037/a0018118

[56] Book: Daniel Kahneman. Thinking, Fast and Slow. Macmillan ISBN 978-1-4299-6935-2; 2011.

[57] Evans JStBT Dual processing accounts of reasoning, judgment and social cognition. Ann Rev Psychology. 2008;59:255–278. 10.1146/annurev.psych.59.103006.093629 18154502

[58] Freeman D Evans N Lister R Gut feelings, deliberative thought, and paranoid ideation: a study of experiential and rational reasoning. Psychiatry Res. 2012;197:119–122. 22406393 10.1016/j.psychres.2011.12.031 PMC3584280

[59] Westermann S Rief W Lincoln TM Emotion regulation in delusion-proneness: deficits in cognitive reappraisal, but not in expressive suppression. Psychol Psychother. 2014;87:1–14. 24497395 10.1111/papt.12000

[60] Colbert SM Peters ER Garety PA Delusions and belief flexibility in psychosis. Psychol Psychother. 2010;83:45–57. 19712542 10.1348/147608309X467320

[61] Garety PA Gittins M Jolley S Differences in cognitive and emotional processes between persecutory and grandiose delusions. Schizophr Bull. 2013;39:629–639. 22499781 10.1093/schbul/sbs059 PMC3627767

[62] Dennis JP Vander Wal JS The Cognitive Flexibility Inventory: instrument development and estimates of reliability and validity. Cog Ther Research. 2010;34:241–253.

[63] Peters ER Moritz S Schwannauer M Cognitive Biases Questionnaire for psychosis. Schizophr Bull. 2014;40:300–313. 23413104 10.1093/schbul/sbs199 PMC3932080

